# Compensatory Regulation of Excitation/Inhibition Balance in the Ventral Hippocampus: Insights from Fragile X Syndrome

**DOI:** 10.3390/biology14040363

**Published:** 2025-03-31

**Authors:** Costas Papatheodoropoulos

**Affiliations:** Physiology Laboratory, Department of Medicine, University of Patras, 26500 Rio, Greece; cepapath@upatras.gr; Tel.: +30-2610-969117

**Keywords:** hippocampus, excitability, GABA, inhibition, excitation/inhibition balance, disorders, fragile X syndrome, brain oscillation, dorsoventral, homeostasis

## Abstract

The excitation/inhibition (E/I) balance is a critical feature of neural circuits, which is crucial for maintaining optimal brain function by ensuring network stability and preventing neural hyperexcitability. The hippocampus exhibits the particularly interesting characteristics of having different functions and E/I profiles between its dorsal and ventral segments. Furthermore, the hippocampus is particularly vulnerable to epilepsy and implicated in Fragile X Syndrome (FXS), disorders associated with heightened E/I balance and possible deficits in GABA-mediated inhibition. In epilepsy, the ventral hippocampus shows heightened susceptibility to seizures, while in FXS, recent evidence suggests differential alterations in excitability and inhibition between dorsal and ventral regions. This article explores the mechanisms underlying E/I balance regulation, focusing on the hippocampus in epilepsy and FXS, and emphasizing the possible mechanisms that may confer homeostatic flexibility to the ventral hippocampus in maintaining E/I balance. Notably, the ventral hippocampus in adult FXS models shows enhanced GABAergic inhibition, resistance to epileptiform activity, and physiological network pattern (sharp wave-ripples, SWRs), potentially representing a homeostatic adaptation. In contrast, the dorsal hippocampus in these FXS models is more vulnerable to aberrant discharges and displays altered SWRs. These findings highlight the complex, region-specific nature of E/I balance disruptions in neurological disorders and suggest that the ventral hippocampus may possess unique compensatory mechanisms. Specifically, it is proposed that the ventral hippocampus, the brain region most prone to hyperexcitability, may have unique adaptive capabilities at the cellular and network levels that maintain the E/I balance within a normal range to prevent the transition to hyperexcitability and preserve normal function. Investigating the mechanisms underlying these compensatory responses in the ventral hippocampus and their developmental trajectories may offer novel insights into strategies for mitigating E/I imbalances in epilepsy, FXS, and potentially other neuropsychiatric and neurodevelopmental disorders.

## Simple Summary

Normal brain function relies on a balanced activation of excitatory (E) and inhibitory (I) neural cells. Disruption in this E/I balance can lead to neurological and neuropsychiatric disorders, including autism spectrum disorder (ASD), fragile X syndrome (FXS), and epilepsy. These disorders are typically associated with an increased E/I ratio and reduced inhibition. The hippocampus, a brain structure involved in fundamental functions such as learning, memory, and emotional regulation, is implicated in several brain disorders, including those mentioned above. Recent experimental findings using a rat model of FXS suggest that the hippocampal segment with the highest inherent tendency toward hyperexcitability—the ventral hippocampus—may possess homeostatic mechanisms capable of compensating for E/I balance alterations during development. Therefore, it is proposed that the ventral hippocampus may serve as a promising model for understanding the spatiotemporal dynamics of adaptive E/I regulation. This could offer valuable insights into potential intervention strategies to correct E/I imbalances in neuropsychiatric and neurodevelopmental disorders.

## 1. Introduction

The balance between excitation and inhibition (E/I) is a fundamental principle governing neural circuit function, ensuring stable network activity, information processing, and cognitive functions [1,2,3,4,5,6,7]. This dynamic interplay between excitatory and inhibitory influences is precisely regulated to maintain effective function of neural circuits, playing a critical role in shaping neuronal responses, controlling network oscillations, and regulating plasticity [3,8,9]. Disruptions in E/I balance can have serious consequences, impairing oscillatory patterns and contributing to neurological disorders such as epilepsy, schizophrenia, autism spectrum disorder (ASD), and fragile X Syndrome (FXS) [4,10,11,12,13,14,15,16,17,18,19,20,21]. The regulation of E/I balance involves a multitude of mechanisms operating at molecular, cellular, and network levels. These range from rapid adjustments in neurotransmitter release and receptor dynamics to slower processes involving gene expression changes and structural remodeling of synapses [3,15,22,23,24,25,26,27,28].

The hippocampus is a complex brain structure crucial for cognitive and emotional processes including spatial navigation, episodic memory, emotional regulation, social behavior, anxiety modulation, and stress response [29,30,31,32,33,34]. Furthermore, the hippocampus is implicated in several neurological and psychiatric disorders including epilepsy, FXS/ASD, schizophrenia, depression, and anxiety disorders [33,35,36,37,38,39]. Given its well-defined circuitry, its crucial role in various cognitive functions, as well as its susceptibility to E/I balance disruptions, the hippocampus serves as an ideal model system for studying the mechanisms of E/I balance regulation and their implications in pathological conditions such as epilepsy and FXS. Interestingly, the dorsoventral axis of the hippocampus shows functional segregation: the dorsal hippocampus is primarily involved in spatial memory and cognition, while the ventral hippocampus is more closely associated with emotional regulation, anxiety modulation, and exhibits greater vulnerability to epilepsy [40,41,42,43] and schizophrenia [44,45]. These functional distinctions are accompanied by molecular and cellular diversity, which may contribute to the differing vulnerabilities of these regions to various disorders [46,47].

Given this context, this review aims to explore the mechanisms of E/I regulation in the hippocampus, focusing on epilepsy and FXS, emphasizing the importance of considering both developmental trajectories and regional specificity when investigating circuit dysfunctions in neurological disorders. We will examine how E/I balance is maintained under normal conditions and how its disruption contributes to pathology, particularly highlighting the differences between the dorsal and ventral hippocampus. Notably, recent studies have revealed intriguing differences in how the dorsal and ventral hippocampus are affected in FXS models. Using *Fmr1* knockout models, it has been shown that the dorsal hippocampus demonstrates hyperexcitability and altered sharp wave-ripples (SWRs) network activity, while, in sharp contrast, the ventral hippocampus exhibits increased GABAergic inhibition that preserves network stability and may contribute to its remarkably reduced tendency for epileptiform activity [48,49,50]. This suggests the presence of homeostatic compensatory mechanisms in the ventral hippocampus that maintain normal network stability by protecting against hyperexcitability.

Understanding the mechanisms underlying these region-specific adaptations may have significant implications for treating these disorders, offering new insights into how E/I balance disruptions can be compensated in the hippocampus and guiding therapeutic approaches for restoring E/I balance in neuropsychiatric and neurodevelopmental conditions. Furthermore, understanding how the ventral hippocampus maintains homeostatic balance is key to clarifying functional segregation along the hippocampal axis and its distinct roles in various disorders.

## 2. The Excitation/Inhibition Balance

Brain functions such as sensory processing, motor actions, and cognition are realized through communication between nerve cells and depend on the coordinated firing of neurons within complex networks, which is essential for the processing and flow of information within these neural networks. Hence, the neuronal networks integrate activity across interconnected neuronal populations, enabling the brain to efficiently process information and accomplish its functions [51,52]. These processes are generally expressed through, or summarized by, the so-called input–output relationship between synaptic inputs and neuronal firing across all time scales [53,54]. Accordingly, the basic objective of neural cell function is to convert inputs, which are generated as synaptic potentials, into outputs represented by action potentials. Furthermore, normal brain function requires that neuronal network activity is maintained stable in the long term to efficiently control moment by moment input–output relationships [27,55,56]. Though long-term stability of overall input–output relationships is a crucial homeostatic requirement for maintenance of consistent behavioral and physiological outputs, neurons and circuits can modulate their input–output properties in response to adaptational demands; for instance, learning and memory processes, or injury and disease can require plastic changes associated with modulation of the input–output dynamics. Such flexibility, which includes maintaining stability and modulating the input–output relationship, is fundamental to ensuring effective behavior and cognitive processes and is achieved through a dynamic balance between excitatory and inhibitory influences at both cellular and circuit levels [8,9,21,26,57].

The balance between excitatory and inhibitory influences, referred to as the excitation/inhibition (E/I) balance or E/I ratio, is a simple approach to describing E/I and represents an emergent dynamic property that regulates activity in neurons and neural circuits. This balance ensures neuronal activity remains within optimal ranges, influencing processes such as information flow, signal amplification, and overall neuronal responsiveness, and preventing excessive excitation or inhibition, which could disrupt normal brain function. Generally, in neuronal networks such as those in the hippocampus, excitation is balanced with inhibition to maintain long-term activity stability, and the precise tunning of excitation and inhibition helps maintain this balance. This proportionality ensures that the network remains functionally stable and effective and experimental evidence supports the idea that balanced excitation and inhibition within neural circuits facilitates their function [3,8,58,59,60]; accordingly, neurons and circuits coordinate their excitatory and inhibitory inputs to establish and maintain a constant E/I ratio.

Maintaining a stable E/I balance is crucial for long-term brain function. Nevertheless, the E/I balance is highly dynamic, indicating that it is subject to rapid changes that are necessary for normal neural processes according to current functional needs [8,9]. In fact, transient, on a millisecond and second basis, and even large changes in the E/I ratio are essential for proper regulation of input–output relationships and normal brain function, and changes in E/I have been shown to accompany behavioral regulation. For instance, differences in the E/I ratio importantly contribute to direct signal flow within and across neuronal circuits [61], while rapid changes in the E/I ratio may contribute to optimally tuning neurons to specific stimuli and shaping their activity pattern in time [9,57]. Still, during memory recall, the brain experiences a brief disruption in the E/I balance, which helps retrieve stored information. This transient imbalance is thought to release memories from inhibitory constraints, enabling their retrieval before the network returns to a stable state [3]. Also, different brain states, such as wakefulness, sleep, or heightened attention, require distinct E/I balances, while the E/I balance also varies across different brain regions, reflecting their specialized functions [5,23,62]. Thus, the temporary dynamic shifts in E/I balance allow the brain to adapt to varying demands by efficiently processing of information and support a variety of functions such as sensory and cognitive processing, memory consolidation, and memory recall [1,2,3,4,5,6,7].

While proper E/I balance ensures stability in neuronal circuits, disruptions in E/I balance can lead to impaired signal processing and are implicated in various neurological and psychiatric disorders. Thus, large enough and/or persistent alterations in the E/I ratio can disrupt dynamics of brain neuronal networks, and dysregulation of E/I balance at the level of brain neural networks is implicated in various neurological and psychiatric conditions, including autism, schizophrenia, and epilepsy, highlighting its importance in maintaining normal brain function [4,12,13,14,16,17,18,21,63,64]. For instance, optogenetically activating inhibitory interneurons or suppressing pyramidal cells in the mPFC was shown to restore social deficits in a genetic mouse model of autism. These results indicate that an E/I imbalance favoring excitation can trigger autism-like symptoms, whereas rebalancing toward inhibition can mitigate these deficits in adult mice [65].

### 2.1. Basic Mechanisms of the E/I Balance

The E/I balance is dynamically modulated through various mechanisms operating across multiple levels of neural organization—at the molecular, cellular, and network levels—and over various time scales—from milliseconds to days (or even longer). These mechanisms converge on maintaining or modulating the E/I balance, ensuring that the E/I balance by showing adaptational flexibility can respond dynamically to immediate needs while maintaining long-term stability. These include neurotransmitter release, receptor and ion channel dynamics, short-term synaptic plasticity, inhibitory interneuron activity, homeostatic plasticity mechanisms (e.g., synaptic scaling and intrinsic plasticity), and synaptic reorganization, all of which contribute to different aspects of neural function [3,15,22,23,24,25,26,27,28].

Mechanisms operating at the molecular and cellular levels, the E/I balance include the regulation of neurotransmitter release, the neurotransmitters’ turnover, and their receptors, and ion channel dynamics. For instance, changing the properties (e.g., conductance), or the expression of ion channels, such as voltage-gated sodium and potassium channels, regulates action potential generation and synaptic transmission, thereby regulating intrinsic excitability of neurons to counteract shifts in network activity. Also, temporal changes in neurotransmitter release probability, receptor trafficking, or receptor activation modulate the E/I ratio across varying time scales. Considering that neurons are the fundamental units for integration of excitatory and inhibitory influences and generate action potentials, the regulation of E/I balance at the single-neuron level is crucial for proper neuronal function and information processing.

At the synaptic and circuitry levels, the E/I balance is primarily regulated through the dynamic interplay between excitatory and inhibitory synaptic inputs [66], while mechanisms of synaptic modulation that regulate the E/I balance include synaptic plasticity, which enhances or reduces synaptic strength in response to activity [67]. Furthermore, adjustments in inhibitory and excitatory neuron populations by neuromodulators can modulate the E/I balance at a slower time scale [68], affecting neuronal network activities like network oscillations. The arrangement of connections of excitatory and inhibitory synapse represents a mechanism of E/I balance at the circuit level [69].

In addition to the fact that the E/I balance is regulated at multiple organizational levels, the underlying mechanisms span a wide temporal range, from fast adjustments (milliseconds to seconds) for immediate functional demands to slow processes (hours to days) for homeostasis and long-term stability and adaptability of neural circuits, allowing for dynamic adjustments across different timescales. This temporal and hierarchical organization of mechanism that regulate E/I balance is essential for balancing the conflicting needs of stability and flexibility in neural circuits [3,22,24,26,27,70,71,72]. Regulation of E/I balance at fast time scale, from milliseconds to seconds or minutes, can be achieved by mechanisms of synaptic modulation such as transient changes in neurotransmitter release probability associated with short-term plasticity phenomena (e.g., synaptic facilitation and depression), fast receptor dynamics (e.g., receptor sensitivity), rapid adjustments of inhibitory synaptic transmission, and fast modulation of ion channel states. Numerous studies have demonstrated that when inhibition closely matches and rapidly tracks excitation on a millisecond timescale in neural networks, it confers significant advantages to the precision, efficiency, and information encoding capacity of neuronal coding mechanisms. Notably, the activity of inhibitory interneurons, such as parvalbumin-expressing (PV) interneurons, plays a crucial role in modulating the E/I balance within neural circuits by regulating the activity of excitatory neurons and ensuring that network activity remains within optimal ranges [9,21,73,74,75]. For instance, high-frequency oscillations, such as gamma rhythms and sharp wave-ripples, rely on rapid localized E/I interactions, which are essential for precise temporal coordination [76].

The modulation of E/I balance over even longer time scales is achieved through mechanisms acting over hours to days or even longer during development and include long-term adjustments in synaptic transmission, intrinsic neuronal excitability, regulation of receptor expression, and remodeling of synaptic connections, as well as the integration of new neurons within a network. One example is the homeostatic control of synaptic strengths that helps restore initial firing rates [24,27,77,78,79]. Developmental processes also play important roles in slow E/I balance regulation; the ratio of excitatory to inhibitory cortical neurons is precisely controlled during development [80,81]. Such homeostatic plasticity is required to compensate for prolonged alterations of activity in a neural circuitry, thereby keeping activity into an optimal physiological range. For instance, in response to prolonged changes in network activity, excitatory synapses may be upscaled or downscaled over hours to days to keep the overall network activity stable [82,83,84]. Also, modulating the strength and number of inhibitory synapses can counterbalance changes in excitatory input, maintaining the E/I balance crucial for proper neural function [24].

While rapid transient E/I balance adjustments, acting over milliseconds and second (e.g., synaptic facilitation), tend to be localized, since they are required for precise computations in restricted spatial domains, more persistent modulation of E/I balance (e.g., neuromodulatory actions), acting over seconds to minutes or longer, often reflects global regulatory mechanisms that stabilizes network function or adapt it to long-term demands. However, there is a spatiotemporal overlap of mechanisms, meaning that the regulation of the E/I balance involves interactions across both spatial and temporal dimensions. Computational work suggests that spatiotemporal dynamics in E/I balance emerge naturally from interactions between fast local inhibitory actions and slower global modulatory influences [85].

**Figure 1:**
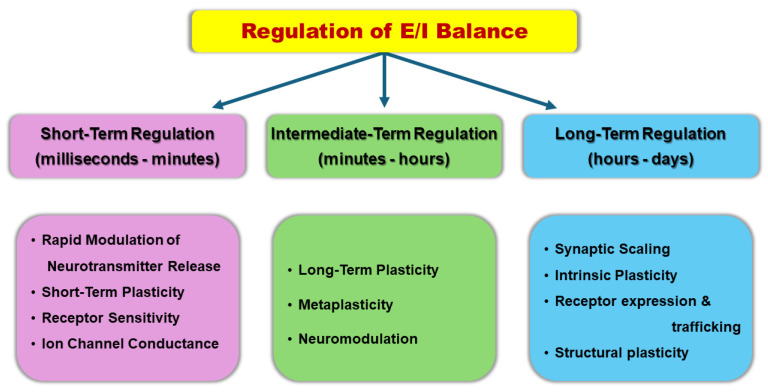
A tentative illustration of key mechanisms regulating E/I balance. The mechanisms are organized based on their primary contributions across different time scales. It should be noted that some mechanisms may extend over wider time windows than this simplified scheme shows.

In conclusion, the regulation of E/I balance involves a complex interplay of fast and slow mechanisms operating at various levels of organization (Figure 1). While rapid changes in the E/I ratio, such as regulation of transmitter release, regulate the input–output relationship in moment-by-moment fashion, more lasting mechanisms of E/I balance regulation, such as intrinsic plasticity, homeostatic plasticity, and structural plasticity, are engaged mainly to adjust the overall excitability of neural networks, ensuring stability in network function over time. Together, these multi-level and multi-timescale processes enable neural circuits to maintain optimal function across a wide range of conditions and experiences.

### 2.2. Homeostatic Regulation of the E/I Balance

The dynamic nature of the E/I balance provides it with the necessary capacity to change in a homeostatic manner, thereby ensuring, as much as possible, the functional adequacy of brain functions and behavior. Indeed, neurons and neural circuits possess intrinsic mechanisms to maintain a balance between excitatory and inhibitory inputs, and accumulating evidence indicates that the E/I balance can be homeostatically auto-regulated, in the sense that a change in one of the two terms of the E/I ratio can lead to a compensatory change in the other ensuring stable function even under conditions of varying input activities [28,64,85,86,87]. Thus, the homeostatic stabilization of input–output relation by virtue of regulation of E/I ratio and intrinsic plasticity can be achieved by adaptive changes either in principal cells or inhibitory interneurons.

Notably, evidence has shown that the E/I balance is dynamically modulated during development and a transient imbalance in the E/I ratio during early development is thought to underline the development of several neurological and neurodevelopmental disorders in adults [4,64,88,89].

For instance, recent evidence suggests that an early, transient increase in the excitability of cortical pyramidal cells during the first two weeks of postnatal development can trigger mechanisms that ultimately reduce the intrinsic excitability of neurons in adult animals. This decreased intrinsic excitability co-occurs with an increased E/I ratio—apparently due to weakened inhibition—and is associated with reduced social behavior [90]. These findings suggest that complex compensatory mechanisms are activated to maintain neural network stability despite early disruptions. Interestingly, additional studies show that early disorder-induced changes in the E/I balance can later lead to homeostatic compensations. For instance, in the tuberous sclerosis factor 1 heterozygote mice and autism spectrum disorder mouse model, a transient hyperexcitability manifested as epileptic seizures, resulted from weakened inhibition [91], occurs during the first 19 postnatal days but not later in the development [92]. Results from another study suggest that a direct disruption of excitatory synaptic inputs leads to a cell autonomous downregulation of inhibitory synaptic inputs, which maintains the E/I balance [93,94]. Nevertheless, the stabilization of the E/I balance is not a reflexive cellular response to any disturbance in synaptic inputs. Rather, it is a targeted mechanism primarily triggered by alterations in excitatory signaling [93]. This specificity is evidenced by the observation that a reduction in GABA_A_-mediated inhibitory inputs does not elicit a corresponding decrease in excitatory inputs [93,95]. It is worth noting that this leading role of excitation in homeostatically regulating the E/I ratio aligns well with developmental patterns where the maturation of inhibitory circuits typically lags behind that of excitatory pathways, as observed, e.g., in sensory cortices [96,97,98].

Interestingly, the enhancement of excitability in brain circuits can lead to compensatory increases in inhibition, and inhibitory interneurons can be targets of homeostatic regulation of E/I balance by virtue of their intrinsic excitability [74,94]. As demonstrated recently in the medial prefrontal cortex of the mouse model of autism (the Tsc2^+/−^ mouse), an increased E/I ratio during the first three postnatal weeks is compensated at later stages (>30 postnatal days) by an elevation of the GABA_A_ receptor-mediated synaptic transmission; yet, a parallel compromised GABA_B_ receptor-mediated tonic inhibition leads to increased neuronal excitability [99]. The results of another study performed in a mouse model of FXS show that an upregulation of cortical GABAergic synaptic transmission at three postnatal weeks may stabilize activity at later development (at six postnatal weeks) [100]. Using optogenetic methods in freely moving animals, compensatory upregulation of GABAergic inhibition has also been observed in cortical circuits following experimentally induced elevation of the E/I ratio, mitigating behavioral deficits [101], thereby offering strong support for the concept that enhancement of excitability in brain circuits can lead to compensatory increases in inhibition. Another example that demonstrates how enhancement of excitability in brain circuits can lead to compensatory increases in inhibition is provided by a study investigating potential changes in the excitability of neuronal networks and individual neurons in the hippocampus elicited by prenatal treatment with valproic acid, in an animal model of autism [102]. These researchers found that the increased intrinsic excitability of single hippocampal neurons and the enhanced network excitability observed in 6-week-old valproic acid-treated rats were later compensated—by 3 months of age—suggesting the development of compensatory inhibitory mechanisms. This provides strong support for the concept that increased excitability in brain circuits can indeed drive compensatory increases in inhibition. Furthermore, the persistence of some alterations until 3 months of age highlights the complex nature of these compensatory processes. Additionally, recent evidence obtained from four genetic models of autism suggests that alterations in the E/I balance may serve as a compensatory mechanism rather than merely reflecting the initial disturbance [103]. Notably, this study reports that a transiently elevated E/I ratio appears to stabilize the overall firing rate of the neuronal network, preventing hyperexcitability during a critical developmental period (15 to 19 postnatal days) associated with autism.

### 2.3. The E/I Balance in Neuropsychiatric and Neurodevelopmental Disorders

Dysregulations of in the E/I balance are typically implicated in major neurological and neuropsychiatric disorders such as epilepsy, schizophrenia, autism spectrum disorders (ASD), Fragile X syndrome (FXS), depression, and anxiety [4,10,11,12,13,14,15,16,17,18,19,20,21], with epilepsy being by far the most typical condition of this disturbance [20,21,104,105,106,107].

Epilepsy is a complex neurological disorder characterized by the occurrence of recurrent seizures, which arise from abnormal electrical activity in the brain, and the mechanisms underlying this activity are complex and multifaceted [106,108,109,110,111]. The causes of epilepsy are remarkably diverse, involving mutations in hundreds of different genes that encode a variety of proteins such as those involved in neuronal development, synaptic transmission (e.g., neurotransmitter receptors), neuronal excitability (e.g., ion channels), and cellular signaling pathways [112,113]. Ultimately, the epilepsy is fundamentally a disorder of disrupted excitation and inhibition, where an imbalance often shifts toward hyperexcitation, leading to seizures [104,114,115]. Epilepsy can also be viewed as a disorder of neural networks where the normal patterns of connectivity and signaling are disrupted, leading to abnormal synchronization of neuronal activity across brain regions, manifesting as seizures [25,116]. Overall, the altered E/I balance contributes to epilepsy symptoms by creating a hyperexcitable neural environment prone to seizures, disrupting normal brain network function, and impacting cognition and behavior.

There is considerable evidence supporting the association of FXS and autism spectrum disorders with altered E/I balance in the brain [13,18,117,118]. Studies indicate that this imbalance is primarily attributed to abnormal glutamatergic and GABAergic neurotransmission in key brain regions, leading to cognitive, sensory, and motor deficits. Specifically, reduced GABA-mediated inhibition has been identified as a key mechanism contributing to hyperexcitability in FXS. Evidence from animal models and human studies supports this association, highlighting the role of interneuron dysfunction in altering E/I balance [119,120].

Schizophrenia is associated with inhibitory deficits, particularly involving GABAergic neurotransmission and altered E/I balance in the brain [121]. The NMDA-hypofunction model also indicates increased excitation in certain patient populations, reinforcing the association between schizophrenia and altered E/I balance [122]. Depression is characterized by reduced GABA and increased glutamate levels leading to altered E/I balance in the brain [123]. Studies indicate that this imbalance contributes to the pathophysiology of major depressive disorder, affecting mood and cognitive functions. Furthermore, studies have shown that individuals with anxiety disorders exhibit alterations in resting-state brain oscillatory patterns compared to healthy individuals. Specifically, in Generalized Anxiety Disorder, there is an imbalance characterized by increased excitatory and decreased inhibitory neurotransmission linked to hyperactivity in brain regions such as the amygdala and prefrontal cortex [19].

## 3. The E/I Balance in the Disordered Hippocampus

As described above, the hippocampus is implicated in a wide range of neurological, neurodevelopmental, and neuropsychiatric disorders, including epilepsy, schizophrenia, autism spectrum disorders (ASD), FXS, depression, anxiety, and stress-related disorders [35,39,124,125,126,127,128,129], and alterations in the E/I balance represent a consistent background of these disorders. Disorder-associated changes in the E/I balance have been most extensively studied in the hippocampus primarily within the context of epilepsy, followed by investigations in FXS/ASD. In contrast, comparatively less focus has been given toward understanding these changes in schizophrenia, depression, anxiety, and other related disorders. Furthermore, numerous findings in FXS/ASD demonstrate parallels with those observed in epilepsy research, particularly within hippocampal circuits, and recent findings have yielded new ideas about the possible homeostatic mechanisms that may work in the ventral hippocampus in a model of FXS to counterbalance the primary effects of this disorder. Therefore, the rest of this review will focus on examining hippocampal mechanisms underlying altered E/I balance specifically in epilepsy and FXS/ASD.

### 3.1. The E/I Balance in the Epileptic Hippocampus

Dysregulation of the E/I balance is the fundamental feature of epilepsy, which is typically characterized by a shift in this ratio towards hyperexcitability, leading to uncontrolled neuronal firing and abnormal synchronization of neuronal networks [104,114,115]. Extensive research has identified molecular (e.g., receptor expression changes), cellular (e.g., interneuron loss), and network-level changes (e.g., axonal sprouting) in the brain that disrupt E/I balance in epilepsy [130]. Primary mechanisms of changes in E/I balance and the associated network hyperexcitability are a heightened excitatory (glutamatergic) activity and/or decreased inhibitory (GABAergic) activity, which play central roles in the pathophysiology of epilepsy [104,116,131]. Disrupted E/I balance can impact synaptic plasticity, a key mechanism for learning and memory, and affect mood regulation and behavior, likely contributing to cognitive impairments often observed in epilepsy patients [132].

The hippocampus, a critical region for memory and learning, is one of the most studied regions in epilepsy since it is highly susceptible to epilepsy/epileptogenesis [129,133,134,135,136,137,138]. In temporal lobe epilepsy, the most common form of focal epilepsy in adults [139,140,141], seizures typically start in, or the seizure’s focus involves, the hippocampus [138,142,143], and hippocampal sclerosis is the most common pathological finding in this type of epilepsy [138,144,145]. The mechanisms underlying epileptogenesis have been extensively investigated in the hippocampus over the past several decades, are multifaceted and involve plasticity changes, ionic imbalances, genetic factors, and neurochemical pathways, which are ultimately expressed as alterations in the E/I balance; these mechanisms are thoroughly discussed in dedicated earlier and recent reviews and treatises (for reviews see: [106,108,109,110,111]; they will not be further discussed here). The objective of this discussion is to highlight and examine more closely specific mechanisms that may be related to compensatory homeostatic attempts, regardless of whether they successfully achieve physiological compensation or fail to restore balance, or they may, in some cases, contribute to the development and progression of epilepsy. Understanding these processes is crucial for developing targeted strategies that can effectively modulate the E/I balance and prevent or mitigate pathological network activity.

In humans, the anterior hippocampus appears to be more epileptogenic and ictogenic than the posterior hippocampus [40,41,42,43]. Furthermore, it has been documented both in vivo [146,147,148,149,150,151,152,153,154] and in vitro [155,156,157,158,159,160,161,162,163,164] that the rodent ventral hippocampus, which corresponds to the human anterior hippocampus, is significantly more susceptible to epileptic/epileptiform activities compared with the dorsal hippocampus. For instance, the ventral hippocampus is identified as the primary site of seizure initiation in animal models of temporal lobe epilepsy [165] and typically shows a higher frequency of epileptiform spontaneous bursting compared to the dorsal hippocampus [48,49,159,161,162,166], and, following seizure activity, it displays more severe and widespread neuronal damage compared to the dorsal hippocampus [154].

Several key structural and functional features of the ventral hippocampus contribute to its heightened epileptogenicity. The ventral hippocampus exhibits stronger projections to amygdalar areas compared with the dorsal hippocampus [167,168], which facilitate seizure propagation, since the amygdala is an epileptogenic area [169,170]. Mutual interconnections between the ventral hippocampus and the entorhinal cortex [171,172], particularly via the temporoammonic pathway [173], can facilitate the spread of synchronous neuronal discharges characteristic of epileptiform activity [174,175,176], creating a substrate for the amplification and sustainment of epileptiform activity. The increased entorhinal input to CA1 neurons [177] and the dramatic loss of feed-forward inhibition in CA1 pyramidal neurons in response to temporoammonic pathway activation [174] renders this loop more prone to sustaining epileptiform activity. Furthermore, the ventral hippocampal commissure tract connects both hippocampi and serves as a functional pathway for seizure propagation [178]. The enhanced connectivity of the ventral hippocampus to seizure-prone regions facilitates the spread of epileptiform activity and increases the likelihood of seizure generalization [165,179]. Additionally, seizures evoked in the ventral hippocampus generalize with fewer stimulations compared to those in the dorsal hippocampus [153].

At the cell and intrinsic network level, the ventral hippocampus CA3 pyramidal neurons have greater dendritic lengths, more complex dendritic arborization, and receive significantly stronger recurrent collateral excitation compared to dorsal CA3 pyramidal neurons [180]. These recurrent connections create positive feedback loops that can amplify and sustain seizure activity once initiated making the ventral hippocampus particularly prone to generating and propagating seizures. Notably, the hippocampal principal cells exhibit increased intrinsic excitability [181,182,183,184,185] and increased ventral hippocampus excitability at the local network level [185,186], although some studies have shown no significant difference [49,187]; it could also be noted in this context that cellular hyperexcitability should be distinguished from circuit hyperexcitability underlying seizures [188]. Furthermore, the glutamatergic NMDA receptors may also contribute to the increased excitability of the ventral hippocampus as has been shown to occur under conditions that promote epileptiform discharges [159,160,164,189].

Interestingly, the ventral hippocampus is vulnerable to the loss of inhibitory interneurons, particularly basket cells, which normally provide crucial inhibitory control. Parvalbumin-expressing interneurons, which include basket cells, axo-axonic cells, and bistratified cells, are among the most vulnerable in temporal lobe epilepsy (TLE) models, such as the intrahippocampal kainate (KA) mouse model [190], and reduced densities of parvalbumin-expressing and somatostatin-expressing interneurons have been found in a model of early life stress (notably, the maternal separation with early weaning model) [191]. The vulnerability of parvalbumin-expressing interneurons and the associated reduction in inhibitory regulation can disrupt their role in controlling network synchrony as observed in neurological and neuropsychiatric disorders, including epilepsy, schizophrenia, and FXS [192,193,194,195,196,197], especially in the ventral hippocampus [191], contributing to seizure initiation and propagation [179]. Furthermore, several studies have provided evidence for reduced GABAergic inhibition in the ventral compared with the dorsal hippocampus [48,49,185,198,199,200], but see also [201,202,203].

The brain attempts to counterbalance disruptions in neural excitability and network function responding to epilepsy or injury with a complex and dynamic set of mechanisms, balancing between compensatory adaptation and potentially detrimental outcomes, and involving molecular, cellular, and network-level mechanisms [188,204,205,206,207,208]. Among the compensatory mechanisms associated with epileptogenesis in the hippocampus, changes in synaptic connectivity, alterations in the expression of ion channels, and alterations in inhibitory circuitry, and neurogenesis are thought to play critical roles in modulating the E/I balance and limiting hyperexcitability [205,207,209]. For instance, functional MRI studies have demonstrated altered patterns of connectivity between the anterior and posterior regions of the hippocampus in individuals with temporal lobe epilepsy, with some individuals showing an increase in connectivity, suggesting it to serve as a compensatory mechanism to counterbalance the impaired function of epileptic regions [210]. Also, the hippocampus undergoes significant reorganization in its circuitry, following injury or seizure activity, that may represent attempts to restore balance. This reorganization can include the sprouting of mossy fibers in the dentate gyrus of the ventral/anterior hippocampus [211,212,213,214] that form new synaptic connections that are not present under normal conditions.

However, these compensatory mechanisms can sometimes lead to maladaptive plasticity, resulting in a network that is more prone to seizures. For instance, recent research suggests that adult-born dentate granule cells born during a critical period after epileptogenic insult may form aberrant excitatory circuits with early-born granule cells [215]. While this could be a compensatory mechanism to replace lost neurons, the newly generated neurons appear to be abnormally integrated into the existing circuit, which might contribute to network dysfunction, demonstrating the complex circuit-level changes that occur in response to hyperexcitability.

The loss of inhibitory interneurons has been tightly connected with the occurrence of epileptic seizures [216,217,218]. Parvalbumin-expressing interneurons have been found to be particularly vulnerable in human temporal lobe epilepsy and in animal models [217,219,220]. The vulnerability of parvalbumin-containing interneurons is thought to be particularly important since these interneurons provide powerful perisomatic inhibition to principal cells, which is crucial to control network excitability [221] and they also play crucial role in generating network oscillations [196], which are important for cognitive functions and are often disrupted in epilepsy. Yet, one of the compensatory responses to hyperexcitability in the hippocampus is an attempt for enhancement of inhibitory mechanisms based on the remaining interneurons [206,207]. In fact, it appears that a reorganization in the GABAergic system can take place in the epileptic hippocampus. For instance, an upregulation of the glutamic acid decarboxylase 67 [190,222], an enzyme responsible for the GABA synthesis, and upregulation of neuropeptide Y [190], has been shown in remaining hippocampal granule cells, with these alterations in the GABAergic system suggested to represent compensatory to the loss of inhibition protecting the neuronal network from further damage [190,223]. Furthermore, extrasynaptic inhibition can be preserved or even increased in the epileptic hippocampus presumably reflecting an attempt to compensate for the impaired synaptic inhibition and protect the hippocampal network [224,225]. Interestingly, in Dravet syndrome—a rare, genetically determined severe form of epilepsy that begins early in life—deficits in GABAergic inhibition are accompanied by a preserved ability of CA1 pyramidal cells to integrate synaptic inputs (i.e., process and combine multiple synaptic signals) [226], suggesting the presence of possible compensatory mechanisms.

### 3.2. The E/I Balance in the FXS Hippocampus

Fragile X syndrome (FXS) is a genetic disorder of the development primarily caused by mutation of the *Fmr1* gene that leads to its inactivation and the loss of fragile X Messenger Ribonucleoprotein (FMRP) [227,228,229]. FXS is a syndrome of intellectual disability and displays a complex phenotype encompassing a number of deficits such as sensory hypersensitivity, hyperarousal, hyperactivity, sleep disturbance, and learning and memory consolidation deficits, anxiety, and social deficits [230,231,232,233,234,235]. FXS is strongly linked to autism spectrum disorder (ASD) since it represents its principal genetic factor [231,233,234,235,236]. Furthermore, FXS and ASD often present with epilepsy as a comorbidity with young FXS individuals displaying increased susceptibility to seizures [16,232,237,238,239,240]. Here, it should be noted that FXS is associated with the interesting paradox that seizures that occur frequently in children and teenagers are drastically reduced or eliminated in adulthood despite the increased excitability in the adult FXS brain [232,240,241,242]. The possible reasons for this will be analyzed in the following sections.

FMRP is widespread expressed in the brain playing basic roles in the regulation of protein synthesis and neuronal activity, and the loss of FMRP in FXS leads to dysregulation of glutamatergic and GABAergic signaling, which are critical for maintaining E/I balance, thereby disrupting the function of neural circuits [243,244,245]. The hippocampus is among the brain regions that are affected by FXS and ASD [39,246,247,248,249,250], with implications for functions such as memory consolidation and learning abilities [251,252]. In animal models of FXS, such as *Fmr1* knockout (KO) mice and rats, hyperexcitability is a prominent feature, suggesting that disruption of the E/I balance in the brain is a fundamental neurobiological substrate of FXS [13,18]. In several animal models and patients with FXS, increased neuronal excitability has been observed in the neocortex [119,253,254,255,256,257,258,259], the dorsal hippocampus [48,49,50,259,260,261,262,263], as well other brain preparations [264,265,266]. This hyperexcitability may arise from multiple mechanisms, including alterations in synaptic function and synaptic connectivity, and dysregulation of ion channel expression and function [16,259,260,267,268,269,270,271,272,273,274,275].

Furthermore, several lines of evidence suggest that a key factor in the altered E/I balance and associated neuronal hyperexcitability in FXS is a reduced or dysfunctional GABA signaling in the brain. This includes alterations in the number and activity of GABAergic cells, the expression of GABA_A_ receptors, GABA content and release, and a delayed development of GABAergic transmission. Impaired GABAergic inhibitory actions have been consistently observed in patients with FXS and animal models of this disorder [119,253,276,277,278,279,280,281,282,283,284,285,286,287,288]. Notably, an imbalance in excitation and inhibition (E/I) can disrupt normal brain oscillations, such as gamma, theta, and sharp wave-ripples, thereby impacting behavior. For instance, the hyperexcitable somatosensory cortex shows sensory hypersensitivity and increased gamma frequency power and synchrony [265,289]. The prefrontal cortex exhibits elevated cellular E/I balance, leading to impaired information processing and social deficits [101]. Auditory cortex shows increased excitatory responses and deficient habituation to repeated stimuli [289], and the thalamus shows disrupted modulation of cortical activity, particularly in theta/alpha frequencies [290]. Patients with FXS show disrupted interneuron firing, increased gamma frequency power, reduced gamma phase-locking to stimulus, and increased sensory sensitivity [291]. Furthermore, the network activity of sharp wave-ripples (SWRs) are among the brain rhythms that are altered in FXS [48,266,292]. SWRs are a hippocampal pattern that occurs during off-line reactivation of specific pyramidal cell assemblies, which are initially formed when the awake animal experiences an event [293,294,295] and are involved in several functions including memory consolidation, decision making, stress and anxiety [295,296,297,298]. These functions are directly or indirectly affected in FXS [231,299,300,301].

A question regarding the effects of FXS, as well as other neurodevelopmental disorders, concerns the temporal progression of these effects on neuronal and network function during development. During early development, the balance of excitatory and inhibitory neurotransmission is critical for proper cognitive and behavioral outcomes. Disruptions in the E/I balance during critical periods of development are thought to play a significant role in establishing hyperexcitable networks [16,267], thereby having decisive implications for brain function at later stages [90]. Evidence suggests that the E/I balance may be disrupted in FXS, leading to increased excitability during brain development. However, the evidence suggests that the disorder-induced changes are not static but evolve over time, and that there is no single homogeneous pattern. Instead, the effects may be region-specific and, during development, can involve homeostatic compensatory mechanisms activated in response to initial alterations. For instance, in a rat model of FXS (FMR-KO), early stages of development showed reduced excitability, with visual responses characterized by lower spike rates before eye-opening. However, by the third and fourth post-natal weeks, signs of mild hyper-excitability began to emerge, indicating a shift towards increased excitability as development progressed [302]. This suggests that while early periods may not exhibit hyper-excitability, later stages do reflect an increase in neuronal firing rates and a disrupted balance between excitatory and inhibitory activity. Also, cortical neurons from neonatal *Fmr1* KO mice show increased epileptiform activity [257].

Accumulating evidence from studies focusing mainly on cortical structures strongly suggests that interneuron dysfunction and altered GABAergic transmission play a significant role in FXS models, particularly during early brain development [253,276,286,303,304,305,306,307,308,309,310,311,312] highlighting the importance of early brain development in FXS pathophysiology [304,311]. Impaired GABAergic transmission has been documented in various brain regions of FXS models. These changes include reduced GABA concentration in the frontal cortex and thalamus of neonatal FXS mice [309], the reduced expression of GABA_A_ receptor subunits [277], and the delayed switch from excitatory to inhibitory GABA signaling in cortical neurons [313]. Also, reduced excitability and firing rates have been documented for parvalbumin- and somatostatin-containing interneurons [286,305]. Suppressing FMRP production in these types of interneurons results in abnormal behavioral traits in adult animals [314]. Furthermore, reduced GABA_A_ receptor-mediated actions but increased GABA_B_ receptor-mediated actions have been described in patients with FXS [119].

It is characteristic that different brain regions can show distinct patterns of interneuron and GABAergic FXS-associated alterations. For instance, specific cortical layers show reduced density in parvalbumin-containing interneurons [276,308], while amygdala neurons show decreased number of inhibitory synapses and GAD65/67 expression (i.e., the GABA synthetizing enzyme) [315]. In addition, while some inhibitory deficits can be partially reversed during development [316], others contribute to lifelong E/I imbalance [276,308,315]. For instance, reduced GABA_A_ receptor δ subunit in neocortex [277], and decreased density of cortical parvalbumin-containing interneurons can lead to behavioral abnormalities such as FXS and anxiety-like behaviors [276,308,317]. This evidence suggests that GABAergic dysfunction in FXS mice follows a non-linear region-specific trajectory during development.

More examples of region-specific abnormal alterations in the GABAergic system during development include GABA concentrations, and structural and functional properties of interneurons, as well as functional properties of GABA receptor-mediated currents. For instance, during the first postnatal week the GABA concentration is reduced in frontal cortex and thalamus [309], and somatosensory cortical interneurons display immature dendritic morphology [304]. Also, the transition from depolarizing to hyperpolarizing GABA current is delayed during the first and second postnatal week [313]. During the third and fourth postnatal weeks interneurons show immature properties [304], and altered activity of parvalbumin-containing interneurons [312]. During the same period profound reduction in both phasic [315] and tonic inhibition [315,318] has been found in the amygdala leading to hyperexcitability. Interestingly, even an increase in GABA release from basket cells has been found to increase excitability in the cerebellum at PND 26-32 [319]. Reduced GABAergic input to cortical principal cells, either in immature or adult *Fmr1* KO mice [320], and reduced GABA release has been shown in the cortex in a model of autism [321]. Furthermore, it has been described that one-month-old mice display dysfunctional inhibitory cortical network [117], and reduced excitation of cortical inhibitory cells [253]. The subiculum of young adult *Fmr1* KO mice shows no change in phasic inhibition, despite a reduction in tonic GABAergic currents [322], offering another example of selective region-specific changes in the GABAergic system. Interestingly, an initial reduction in inhibition can lead to homeostatic responses that can partially restore neural circuit function [323], and alterations suggestive of homeostatic mechanisms can occur at the molecular level very transiently during brain development (from PND 18 to 19) [311].

Research indicates that also in the hippocampus, excitability and the E/I ratio undergo significant changes during early development in individuals with FXS, both in human studies and animal models. Increased excitability of the dorsal hippocampus has also been suggested for immature and young (aged 3–8 weeks) *Fmr1* KO mice [270]. The CA3 hippocampal region of *Fmr1* KO mice shows hyperexcitability at the age of PND 19-24 due to downregulation of SK channels [261]. Increased intrinsic excitability of the dorsal CA1 hippocampal pyramidal cells has been demonstrated in adult *Fmr1* KO mice [260]. In behavioral studies involving *Fmr1* ^−^/^y^ rats, it was found that while initial firing rates of CA1 hippocampal pyramidal neurons were similar to wild-type rats, these neurons did not exhibit the same experience-dependent changes over time. This lack of adaptation suggests an underlying increase in excitability that is not effectively modulated by environmental experiences [324]. Furthermore, recent data suggest that the hyperexcitability of CA1 hippocampal neurons may represent a mechanism that operates to compensate for an initial disturbance (reduction) in neuronal activity during the postnatal development of *Fmr1* KO mice [263]. Together, these data show that neuronal excitability is increased in the adult dorsal hippocampus. A delay in the developmental switch of GABA_A_ receptor polarity, from depolarizing to hyperpolarizing, has been observed in the hippocampal [267] and cortical neurons [313], that may exacerbate excitability during critical developmental periods.

As described earlier, *Fmr1* KO animals lack FMRP, a protein crucial for regulating protein synthesis and synaptic function, which is also involved in regulating the function of GABA_A_ receptors in the hippocampus [325]. However, inhibitory influences have been less extensively studied in the FXS hippocampus compared to other brain regions. Immediately after birth, there is a severe impairment of giant depolarizing potentials (GDPs) in the CA3 hippocampal region in an animal model of idiopathic autism [326]. This is associated with increased GABAergic neurotransmission and reduced neuronal excitability, despite GABA’s depolarizing action at this stage. Phasic and tonic inhibition, as well as the expression of α2, β1, and δ GABA_A_ receptor subunits, have been found to be reduced in CA1 hippocampal pyramidal neurons from young adult *Fmr1* KO mice [279]. Additionally, altered expression of β2, β3, or non-specific β GABA_A_ receptor subunits has been reported in the hippocampus of young adult Fmr1 KO mice [281,327]. Deficits in synaptic transmission at excitatory synapses onto CA1 inhibitory interneurons [328] and deficient signaling via presynaptic GABA_B_ receptors at Schaffer collaterals [284,329] have been documented in immature Fmr1-deficient mice. Finally, reduced GABA but elevated α1 subunit have been observed in the hippocampus of adult mice in a model of autism [330]. Notably, these studies did not distinguish between different segments of the hippocampus.

## 4. Dorsoventral Organization of the Hippocampus

The hippocampus is a brain structure located in the medial temporal lobe of humans, extending in the anterior–posterior direction, while in rodents it extends along the dorsoventral or septotemporal axis. The hippocampus is recognized for its deep implication in spatial navigation, episodic memory, and memory consolidation [331,332,333,334]. Furthermore, the hippocampus is involved in a plethora of other brain functions, including emotional processing, stress response, social behavior, fear learning, and anxiety [29,30,31,32,33,34]. Given the critical role of the hippocampus in multiple brain functions, it is not surprising that it is also implicated in various neurological, neuropsychiatric, and neurodevelopmental disorders, like epilepsy, Alzheimer’s disease, schizophrenia, depression, FXS, autism spectrum disorder (ASD), post-traumatic stress disorder, and other anxiety-related conditions [33,35,36,37,38,39].

The hippocampus is internally organized into a basic trisynaptic circuit that processes information through distinct sequentially connected subregions, namely, the dentate gyrus (DG), CA3, CA1, and subiculum. The entorhinal cortex is the main brain region from which the hippocampus receives input and sends its output [335]. This main feedforward architecture of the hippocampal circuitry is modulated by a network of GABAergic inhibitory interneurons, which comprise about 20% of hippocampal neurons and are essential for maintaining the E/I balance, ensuring proper information processing and precise temporal coding and network stability while preventing pathological states such as hyperexcitability [336]. Disrupted E/I balance underlies hyperexcitability in disorders like epilepsy and FXS. Thus, hippocampal computation relies on finely tuned interactions between glutamatergic pathways and specialized interneuron subtypes, with parvalbumin-containing interneurons serving as master regulators of network dynamics [336,337]. This interplay between excitatory and inhibitory neurons is crucial for network oscillations, such as theta rhythm, gamma rhythm, and sharp wave-ripples, which are associated with various cognitive functions such as spatial coding, memory consolidation, and adaptive plasticity while preventing pathological activities. Disruptions in the E/I balance can impair these oscillations, leading to cognitive deficits and increased susceptibility to neurological disorders.

### 4.1. Functional Specialization Along the Hippocampus

Traditionally, the hippocampus is divided based on its location along the longitudinal axis. In rodents, the dorsal hippocampus is positioned at the top and the ventral hippocampus at the bottom. In humans and primates, the hippocampal head and tail are referred to as the anterior and posterior hippocampus, corresponding to the ventral and dorsal hippocampus in rodents, respectively. An intermediate segment of the hippocampus has also been proposed, exhibiting distinct properties [338,339,340]. However, for simplicity, this article will refer only to the dorsal and ventral segments of the hippocampus. Based on functional, connectivity patterns, gene expression profiles, and cellular properties—among other differences along the longitudinal (i.e., dorsoventral or septotemporal) axis—a distinction can be made between the dorsal/posterior and ventral/anterior hippocampus [30,31,46,47,168,340,341,342,343,344,345]. This segmentation is crucial for understanding hippocampal function, as each region appears to play distinct roles in cognition, memory, and emotion.

In general, while the dorsal/posterior hippocampus is primarily involved in spatial and cognitive processing, the ventral/anterior hippocampus plays a greater role in emotional, motivational, and anxiety-related responses. Nevertheless, this distinction represents a somewhat oversimplified view of the specific roles of each hippocampal segment. Notably, the dorsal hippocampus is primarily involved in processing spatial information and navigation, helping animals and humans orient themselves in complex environments. It plays a fundamental role in episodic memory formation, particularly in memory related to spatial contexts, by associating specific contexts with experiences, such as linking particular environments with aversive stimuli (fear conditioning) and facilitating the detailed recollection of specific events [346,347,348,349]. Based on the extensive connections with the amygdala, prefrontal cortex, and its interaction with the hypothalamic–pituitary–adrenal axis, the ventral/anterior hippocampus is involved primarily in regulating emotional and affective processing, including fear and anxiety modulation, stress regulation, and some forms of memory processing [31,33,34,340,350]. Notably, the ventral/anterior hippocampus supports associative memory, allowing individuals to connect various experiences thereby helping generalize memories across different contexts, an ability particularly important for adapting to new but similar situations [351]. Additionally, the anterior hippocampus has been shown to play a role in social cognition, specifically in encoding and recalling information related to relationships and social interactions [32,352,353]. Interestingly, impairments in social cognition are associated with several neuropsychiatric and neurodevelopmental disorders, many of which affect how individuals perceive, interpret, and respond to social information.

The striking dorsoventral diversification at the molecular, cellular, and circuitry levels (see reviews by [46,47]) suggests that interactions across different levels of organization shape the specific functional roles of the hippocampus in behavior. It is also important to emphasize that a significant distinction between the dorsal and ventral hippocampus involves their E/I balance profiles. Perhaps the most striking difference is the greater tendency of the ventral hippocampal neural network toward hyperexcitability, which manifests as increased vulnerability to epilepsy [40,41,42,43]. The distinct E/I profile of the two hippocampal segments may confer specific adaptive capabilities. As described below, various features of the hippocampus, from its unique network organization to its involvement in multiple disorders, have established the hippocampus as a model for studying the mechanisms of E/I balance, both under physiological conditions and in the context of various disorders.

### 4.2. Dorsoventral Circuit Diversification in the FXS Hippocampus

**Figure 2:**
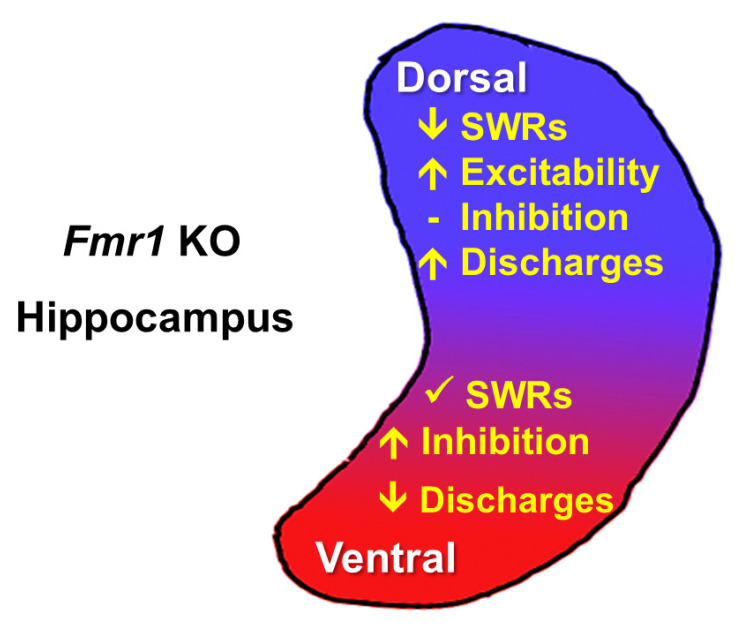
Schematic figure illustrating the main effects of FXS in the dorsal and ventral hippocampus of adult rats. In the *Fmr1* KO rat model of FXS, the loss of FMRP is associated with disrupted sharp wave-ripple activity (SWRs), increased network excitability, and a higher frequency of epileptiform discharges without changes in GABAergic inhibition. In sharp contrast, the ventral hippocampus of *Fmr1* KO adult rats shows normal SWRs, enhanced GABAergic inhibition, and a reduced frequency of epileptiform discharges, suggesting the activation of compensatory mechanisms that maintain normal network activity.

Recent studies have investigated the excitability and inhibition in the CA1 region of the dorsal and ventral hippocampus of adult *Fmr1* KO rats and found different profiles along the hippocampal dorsoventral axis [48,49,50] (Figure 2). Findings indicate that the dorsal hippocampus exhibits increased excitability, as evidenced by enhanced evoked population responses and a higher frequency of epileptiform discharges in slice preparations. Additionally, the pattern of SWRs in the dorsal hippocampus is altered in *Fmr1* KO rats [48,266,292]. These alterations in the excitability of the dorsal hippocampus occur without significant changes in GABA_A_ receptor-dependent signaling [48,49], or the number of GABAergic neurons [276], suggesting that the heightened excitability is not due to deficits in inhibitory neurotransmission. Furthermore, this implies that inhibitory function in the dorsal hippocampus may be insufficient to counteract the increased excitability.

Interestingly, the ventral hippocampus, which also exhibits signs of enhanced excitability, presents a distinct profile of GABAergic transmission, SWRs, and susceptibility to epileptiform discharges. In particular, the GABAergic system in the ventral hippocampus of adult *Fmr1* KO rats is upregulated in terms of the effectiveness of inhibition of CA1 pyramidal cell firing and the expression of the α1 subunit containing GABA_A_ receptors. Remarkably, the ventral hippocampus of these rats shows resistance to epileptiform discharges, which strongly contrasts with the heightened tendency of the ventral hippocampus to epileptic/epileptiform activities in wild-type rats. As has been described above, the ventral hippocampus is the brain region most susceptible to seizures. Accordingly, under conditions that boost neuronal excitability such as FXS, it is vital for this region to be able to counterbalance the initially enhanced excitability in a way that ensures that it can continue to function normally. Accordingly, the upregulation of the GABAergic transmission in the adult ventral hippocampus may be related to the reduced susceptibility to epileptiform discharges observed in this segment of the hippocampus, explaining to some extend the reduced appearance of seizures in adult patients with FXS [232,240,241,242]. Although the pattern of seizures in FXS mostly resemble benign childhood epilepsy with centrotemporal spikes [237,354], an involvement of the hippocampus in childhood epilepsy can be possible [355] considering the anatomical and functional alterations of the FXS hippocampus and the fact that FMRP is highly expressed in the hippocampus [247,267,354,356,357]. Therefore, this enhanced inhibition in the ventral hippocampus may represent a homeostatic adaptation to prevent hyperexcitability despite the loss of FMRP.

FXS is associated with altered theta and gamma oscillations in the hippocampus [358], while the loss of FMRP also affects sharp wave-ripples (SWRs) [48,266,292]. SWRs are a complex pattern that are associated with a transiently enhanced excitability [359] involving a complicated interaction of principal cells with specific types of GABAergic interneurons and especially parvalbumin-containing cells [360,361,362]. Though the occurrence of SWRs is associated with a transient enhancement of neuronal excitability, the physiological generation of SWRs, however, appears to require a background of neuronal activity where excitation is well balanced by inhibition [295,363,364]. This condition—of well-tuned E/I balance—may facilitate the regulation of transient change in E/I balance associated with the occurrence of SWRs. Research has shown that deviation of the background E/I balance, to either direction, can disrupt the ability of the hippocampal circuitry to generate normal SWRs activity [365,366,367,368,369,370,371]. Also, recent data show that the development of inhibition promotes this activity [372], and SWRs are also favored under conditions of simultaneous increase in excitation and inhibition in the hippocampal network [186,372].

Therefore, it is conceivable that the maintenance of normal SWRs is critically supported by the enhancement of GABAergic inhibition in the ventral hippocampus of adult *Fmr1* KO rats. In contrast, enhanced excitability without corresponding inhibition may be crucial in altering SWR properties in the dorsal hippocampus of these rats, potentially implicating it in the symptomatology of FXS. This is particularly relevant given that the E/I balance in the hippocampus plays a crucial role in shaping the behavioral phenotypes observed in FXS. For instance, hyperexcitability in the dorsal hippocampus of *Fmr1* KO animals appears to lead to altered SWRs, which are essential for memory consolidation and may contribute to the learning difficulties and intellectual disability characteristic of FXS [Liu-2022] [16]. Furthermore, considering the relationship between SWRs and stress and anxiety [296,297,373], changes in SWRs in the dorsal hippocampus may be linked to relevant symptomatology in individuals with FXS. On the other hand, the preservation of SWRs, combined with increased inhibitory signaling and a reduced tendency for hyperexcitability in adult *Fmr1* KO rats, despite the overall hyperexcitability observed in FXS, may positively influence anxiety-like behaviors and emotional regulation in individuals with FXS. This is particularly relevant given the strong association of the ventral/anterior hippocampus with anxiety and emotional processing [31,33].

The differential effects of FMRP loss along the hippocampal axis highlight the complexity of excitability changes in FXS. While hyperexcitability is a hallmark feature, compensatory mechanisms, such as increased inhibition in the ventral hippocampus, suggest region-specific adaptations. Relevant to this, previous evidence has shown that FXS is associated with neurobiological changes that are specific to certain brain regions and even cell types [259,284,374,375,376]. The potential operation of compensatory mechanisms in the ventral hippocampus is not surprising, given the broad range of functions it supports and the critical need to maintain these functions within a normal range. This adaptation could be considered a form of flexibility in this brain region, helping to preserve its functionality, and preventing a shift toward hyperexcitability, which could affect multiple brain functions.

Understanding how the E/I imbalance in the dorsal hippocampus and the apparent homeostatic adaptations in the ventral hippocampus of FXS models impact cognitive and emotional processes, sensory responsiveness, and seizure susceptibility is crucial for developing targeted therapeutic strategies aimed at restoring E/I balance and improving functional outcomes in FXS. Furthermore, investigating the mechanisms by which the hyperexcitability tendency of the ventral hippocampus transforms into resistance to epileptic discharges may provide valuable insights for developing novel approaches to treat seizures in children with FXS and hippocampal epilepsy in general. Additionally, these dorsoventral differences in the adult FXS condition underscore the importance of considering both developmental timing and regional specificity when studying circuit dysfunctions in neurodevelopmental disorders [377]. Further research is needed to clarify the developmental trajectories of excitability in the dorsal and ventral hippocampus in FXS, identify the early stages of these changes, and determine how they contribute to behavioral phenotypes such as anxiety, memory deficits, and seizure susceptibility observed in FXS.

## 5. Conclusions

The study of E/I balance in the hippocampus, particularly in the context of epilepsy and FXS, reveals the complex and dynamic nature of neural circuit regulation in health and disease. The distinct profiles of E/I alterations observed along the dorsoventral axis of the hippocampus in these conditions highlight the importance of considering regional specificity in neuroscience research and therapeutic development. Specifically, the ventral hippocampus appears as a dynamic neural circuit with remarkable flexibility in maintaining E/I balance. Recent evidence from *Fmr1* knockout models of FXS highlights the distinct adaptive mechanisms that may operate in the ventral hippocampus, where enhanced GABAergic inhibition appears to counterbalance increased excitability and prevent pathological network activity, thereby crucially contributing to maintaining normal activity patterns. The intriguing apparent adaptation seen in the ventral hippocampus of adult FXS models emphasizes the importance of region-specific therapeutic approaches and may represent a remarkable example of homeostatic flexibility in the brain that offers important insights into how the ventral hippocampus could serve as a model for studying and treating neuropsychiatric and neurodevelopmental disorders. Identifying the developmental stage(s) during which potential homeostatic mechanisms are activated leading to the regulation of E/I balance and the restoration of normal function in the ventral hippocampus could effectively contribute to both the understanding of processes occurring during development in FXS and other neurodevelopmental disorders. Understanding these developmental stages could aid in the development of ideas for designing interventions during development that could prevent the manifestation of at least some symptoms associated with E/I balance disruption.

In future studies, it is of interest to investigate the developmental trajectories of these regional E/I alterations, particularly in FXS. These studies should focus on the molecular and circuit-level processes that enable this compensatory flexibility in the ventral hippocampus during the development, including intrinsic properties of principal neurons, various aspects of GABAergic transmission, as well as the local network’s propensity to exceed the threshold for hyperexcitability. Since these mechanisms are likely developed during early postnatal periods and continue into adulthood, future research should include both early postnatal development (first three weeks), and the period of adolescence (up to eight weeks). Understanding the spatiotemporal dynamics in E/I balance adaptive regulation within the ventral hippocampus could provide valuable insights into potential compensatory strategies to maintain functionality in the face of genetic disruptions and improving outcomes for individuals with hippocampal dysfunction, thereby having broader implications for understanding and treating other neuropsychiatric and neurodevelopmental disorders characterized by E/I imbalance. Underscoring the need for a region-specific approach to studying brain disorders and developing treatments, it is proposed that the ventral hippocampus offers a promising framework for the development of interventions that could adjust E/I imbalances in the brain.

## Data Availability

Not applicable.
